# Supplementary material for: Immune Reconstitution Inflammatory Syndrome in People Living with HIV Who Presented with Interstitial Pneumonitis: an Emerging Challenge in the Era of Rapid Initiation of Antiretroviral Therapy
Source: Microbiol Spectr. 2023 Mar 6;11(2):e04985-22. doi: 10.1128/spectrum.04985-22 (PMC10100876; doi:10.1128/spectrum.04985-22)
Supplement: Supplemental file 1 — Tables S1 and S2. Download spectrum.04985-22-s0001.pdf, PDF file, 0.6 MB [file spectrum.04985-22-s0001.pdf]

Supplementary Table 1. **Sensitivity analysis of theassociation between the interval between the diagnosis of IP and ART initiation with paradoxical IRIS in people living with HIV who presented with interstitial pneumonitis**[a]

Variable	Univariate		Multivariate	
	HR (95% CI)	*p* Value	Adjusted HR (95% CI)	*p* Value
ART initiation <1 days of IP diagnosis, n=20	1.01 (0.37-2.74)	0.99	0.90 (0.33-2.50)	0.85
ART initiation <2 days of IP diagnosis, n=33	**3.62 (1.52-8.65)**	**0.004**	2.35 (0.94-5.88)	0.07
ART initiation <3 days of IP diagnosis, n=44	**2.58 (1.05-6.32)**	**0.04**	2.12 (0.82-5.48)	0.12
ART initiation <4 days of IP diagnosis, n=52	**3.76 (1.27-11.12)**	**0.02**	**3.18 (1.06-9.55)**	**0.04**
ART initiation <5 days of IP diagnosis, n=57	**6.54 (1.53-28.00)**	**0.01**	**4.91 (1.12-21.55)**	**0.04**
ART initiation <6 days of IP diagnosis, n=61	**11.32 (1.52-84.21)**	**0.02**	**9.27 (1.21-70.79)**	**0.03**
ART initiation <7 days of IP diagnosis, n=67	**7.84 (1.05-58.32)**	**0.04**	**7.95 (1.04-60.90)**	**0.05**
ART initiation <8 days of IP diagnosis, n=69	6.84 (0.92-50.91)	0.06	7.32 (0.96-55.70)	0.06
ART initiation <9 days of IP diagnosis, n=75	4.23 (0.57-31.49)	0.16	4.86 (0.64-36.88)	0.13

Abbreviations: 95% CI, 95% confidence interval; ART, antiretroviral therapy; IP, interstitial

pneumonitis; HR, hazard ratio

[a]The covariates of CD4-to-CD8 ratio <0.1, Interval change of PVL in a month and CMV viremia were included in the multivariable model.

Supplementary Table 2. Factors associated with respiratory failure in people living with HIV who presented with interstitial pneumonitis

Variable	Univariate		Multivariate	
	HR (95% CI)	*p*-value	Adjusted HR (95% CI)	*p*-value
Age at admission, per 1-year increase	0.97 (0.91-1.04)	0.46	-	-
Obesity (BMI >27 kg/m^2)	**6.38 (1.44-24.87)**	**0.01**	**40.8 (2.70-616.87)**	**0.01**
Recent CD4 count, per 10-cell/mm^3 increase	0.92 (0.76-1.12)	0.41	-	-
Recent CD8 count, per 10-cell/mm^3 increase	0.99 (0.97-1.01)	0.29	-	-
CD4-to-CD8 ratio <0.1	0.97 (0.28-3.36)	0.97	-	-
Absolute neutrophil count, per 10-cell/mm^3 increase	**1.003 (1.001-1.005)**	**0.002**	**1.01 (1.00-1.01)**	**0.01**
C-reactive protein, per 1-mg/dL increase	0.96 (0.75-1.24)	0.78	-	-
Serum LDH, per 1-IU/L increase	**1.01 (1.00-1.01)**	**0.02**	0.99 (0.99-1.00)	0.63
Latest PVL, per 1-log_{10}	1.36	0.52	-	-

copies/ml increase	(0.53-3.46)			
Interval between the diagnosis of IP and ART initiation, per 1-day increase	1.06 (0.97-1.15)	0.19	-	-
ART initiation <7 days of IP diagnosis	0.62 (0.16-2.39)	0.49	-	-
Prednisone-equivalent dose in the first 5 days, per 1-mg/kg/day increase	1.00 (0.99-1.00)	0.23	-	-
INSTI-based regimen	1.28 (0.27-6.02)	0.76	-	-
CMV viremia	0.60 (0.17-2.14)	0.43	-	-

Abbreviations: 95% CI, 95% confidence interval; ART, antiretroviral therapy; BMI, body-mass index; CMV, cytomegalovirus; HR, hazard ratio; INSTI, integrase starnd-transfer inhibitor; LDH, lactate dehydrogenase; PVL, plasma HIV RNA load.
